# Clinical features of influenza-associated pulmonary aspergillosis: a retrospective multicenter cohort study

**DOI:** 10.3389/fcimb.2025.1648547

**Published:** 2025-10-15

**Authors:** Jia-Xin Shi, Xiang-Rong Shao, Peng Wu, Xiang Wang, Kui Sheng, Qi-Chao Chen, Feng Jin

**Affiliations:** ^1^ Department of Respiratory and Critical Care Medicine, Affiliated Hospital of Yangzhou University, Yangzhou University, Yangzhou, Jiangsu, China; ^2^ Department of Respiratory and Critical Care Medicine, Jiangdu People’s Hospital, Yangzhou, Jiangsu, China; ^3^ Department of Respiratory and Critical Care Medicine, Nanjing Drum Tower Hospital Group Yizheng Hospital, Yangzhou, Jiangsu, China; ^4^ Department of Respiratory and Critical Care Medicine, Gaoyou People’s Hospital, Yangzhou, Jiangsu, China

**Keywords:** influenza, influenza-associated pulmonary aspergillosis (IAPA), Galactomannan (GM) test, microbiology rapid on-site evaluation (M-ROSE), targeted next generation sequencing (tNGS)

## Abstract

**Rationale:**

Influenza-associated pulmonary aspergillosis (IAPA) is a great clinical challenge, which has high morbidity and mortality in severe influenza infections. Unlike invasive pulmonary aspergillosis (IPA), IAPA often occurs in immunocompetent hosts. That IAPA lacks of specific manifestations makes it difficult to diagnose and causes a high mortality rate, which is becoming challenges toclinicians. Exploring the clinical characteristics of IAPA in depth is of great significance for guiding clinical practice.

**Objectives:**

To determine the clinical features of IAPA in order to support appropriate clinical management of this public health threat.

**Methods:**

A retrospective multicenter cohort study was conducted. The clinical characteristics, risk factors, diagnostic methods, treatment, and prognosis data of the participants were analyzed.

**Results:**

Diabetes and lymphopenia were important risk factors for IAPA. Pulmonary imaging showed that IAPA patients had more nodular lesions in the lungs, and a higher proportion of them were accompanied by cavitary lesions. The galactomannan (GM) test in bronchoalveolar lavage fluid (BALF) showed high sensitivity and specificity for diagnosing IPA. The positive rate of *Aspergillus* culture was relatively low, while microbiology rapid on-site evaluation (M-ROSE) could effectively detect mould with high specificity. The targeted next generation sequencing (tNGS) had important value in detecting *Aspergillus* with a sensitivity of 100% and a specificity of 86.7%. Both IAPA and Non-IAPA patients had a higher rate of co-infections, with a significantly higher co-infection rate of atypical pathogens in the IAPA group compared to the Non-IAPA group. The average treatment course for IAPA patients in the present study (32.2 ± 4.8d) was greatly shorter than the course specified in the IDSA (2016) guideline. The neutrophil to lymphocyte ratio (NLR) could effectively predict the prognosis of IAPA patients (cutoff value of NLR was 17.70, corresponding to a sensitivity of 0.87 and a specificity of 0.97).

**Conclusions:**

Diabetes and lymphopenia were important risk factors for IAPA. The comprehensive application of serum and BALF GM, M-ROSE and tNGS could significantly improve the detection rate of *Aspergillus*.

## Introduction

Influenza-associated pulmonary aspergillosis (IAPA) has emerged as a significant clinical challenge, particularly among patients with severe influenza infections. IAPA was first described in 1952 and was considered to be a rare condition before but is now recognized as a significant contributor to morbidity and mortality, with reported incidence rate of 19% in ICU patients and mortality of 50% (Schauwvlieghe et al., 2018). Invasive pulmonary aspergillosis (IPA) was often seen in severely immunocompromised hosts (Patterson et al., 2016), and the mortality was reported to be up to 30% (Colombo et al., 2017), which could be much higher in critically ill patients (Zubovskaia and Vazquez, 2025). Unlike IPA, which is commonly found in immunocompromised hosts, IAPA often occurs in immunocompetent patients, challenging traditional diagnostic paradigms (Lu et al., 2024). Influenza virus has always been a major threat to humans, with high variability and widespread susceptibility (Jain et al., 2025). During the flu season, the increased pathogenicity caused by mutations can lead to many critically ill patients, increasing the chances of various secondary infections (Lyons and Lauring, 2018). IAPA, due to lack of specificity in clinical manifestations, is difficult to diagnose early and has a high mortality rate, which is becoming a major clinical challenge. How to quickly diagnose IAPA and provide appropriate treatment is the key to improve prognosis. In recent years, there have been many reported cases of influenza worldwide, which include many cases of IAPA (Feys et al., 2024). Understanding clinical features, common pathogens of co-infection and searching for biomarkers that predict the prognosis of IAPA can help improve risk identification for patients and give appropriate management. Therefore, we conducted this retrospective multicenter cohort study to explore the above issues.

## Methods

### Study design and patients

A retrospective multicenter cohort study was conducted in the Department of Respiratory and Critical Care Medicine of Affiliated Hospital of Yangzhou University, Jiangdu People’s Hospital, Nanjing Drum Tower Hospital Group Yizheng Hospital and Gaoyou People’s Hospital between January 2024 and May 2025. The study was approved by the local ethics committees (2025-YKL02-(K01)).

The patient inclusion criteria were as follows: (1) patients with age ≥18 years; (2) patients with a positive influenza polymerase chain reaction (PCR) from nasopharyngeal swab or bronchoalveolar lavage fluid (BALF); (3) patients had pulmonary infiltrates on chest imaging. The diagnostic criteria for invasive pulmonary aspergillosis (IPA) were based on the EORTC/MSGERC (2020), IDSA (2016), and FUNDICU (2024) guidelines (Bassetti et al., 2024; Donnelly et al., 2020; Patterson et al., 2016). Proven IPA needs a positive *Aspergillus* in sterile body fluids or tissues. The probable IPA requires the combination of (1) host factors; (2) clinical symptoms; (3) chest CT showing lesions, infiltrative shadows, or cavities and (4) microbiological evidence, such as positive for *Aspergillus* culture or PCR, single Galactomannan (GM) test ≥1.0, or single serum GM ≥0.7 with BALF GM ≥0.8. The IAPA patients were reviewed and consensus was achieved by two senior pulmonologists according to the host risk factors, clinical symptoms, chest imaging, laboratory test, and response to treatment. Patients were excluded if the clinical information was insufficient. Non-IAPA were the patients with influenza but without pulmonary aspergillosis.

Respiratory co-infection was defined as (1) detection of clinically relevant bacterial or fungal pathogen in BALF or positive blood culture which was treated using antibiotics or antifungals, respectively; (2) a positive detect for Cryptococcal capsular polysaccharide in blood, or positive GM in serum or BALF.

### Statistical analysis

All statistical analysis was carried out with SPSS 26.0 software (Chicago, USA). The numerical measurements were given as mean ± standard deviation. Receiver operating characteristic (ROC) curve analysis was performed and the areas under the working characteristic curve (AUC) values were displayed, including a 95% CI. An independent sample t-test was employed to analyze the differences between groups and the Chi-square test was used to compare counting data. Odds ratio (OR) was calculated for the baseline data that differed between the two groups. Two-tailed probability values of < 0.05 were considered statistically significant. The pie chart and bar chart were made in Excel spreadsheet (version 2010).

## Results

### Patient characteristics

This study enrolled 140 patients which were stratified into two groups: Non-IAPA (n=98) and IAPA (n=42). Baseline characteristics and clinical features between groups were systematically compared, and no significant differences between both groups were observed in age, sex, influenza subtype, or most comorbidities, except for diabetes (**Table 1**). ​​Diabetes was significantly more prevalent in IAPA patients (35.7%, 15/42) than that in Non-IAPA patients (17.3%, 17/98; p = 0.018, OR=2.65). Other comorbidities, including ​​COPD​​, ​​cardiovascular disease, ​​chronic kidney disease, and ​​solid cancer​​, showed no significant differences between groups. Rare conditions such as ​​hematologic malignancy​​ and ​​hepatitis were similarly distributed. ​​Lymphopenia​​ at influenza diagnosis was remarkably higher in IAPA patients (66.7%, 28/42) compared to Non-IAPA patients (39.8%, 39/98; p = 0.004, OR=3.03). ​​​​Chemotherapy​​ exposure was rare and limited to Non-IAPA patients. Recent ​​corticosteroid use​​ (within 28 days) was 4.8% (2/42) of IAPA patients and 3.1% (3/98) of Non-IAPA patients. Neutropenia​​ was rare in both groups. ​​CURB-65 scores​​, indicating pneumonia severity, were comparable between groups. Influenza A​​ was the dominant subtype in both groups, while ​​Influenza B​​ was less common. The baseline data indicated that Diabetes​​ and ​​lymphopenia​​ emerged as significant risk factors for IAPA, with Diabetes prevalence doubling in the IAPA group.

**Table 1:** Baseline characteristics of Non-IAPA and IAPA patients.

Characteristics	Non-IAPA (*n* =98)	IAPA (*n* =42)	*p va*lue	OR
Mean age, years (SD)	67.3 (15.2)	65.9 (10.8)	0.096	
Male sex (%)	59 (60.2)	30 (71.4)	0.206	
COPD (%)	14 (14.3)	8 (19.0)	0.478	
Diabetes (%)	17 (17.3)	15 (35.7)	0.018*	2.65
Hepatitis (%)	2 (2.0)	1 (2.4)	0.899	
Chronic kidney disease (%)	4 (4.1)	1 (2.4)	0.619	
Cardiovascular disease (%)	15 (15.3)	9 (21.4)	0.378	
Hematologic malignancy (%)	1 (1.0)	0 (0)	0.511	
Solid cancer (%)	5 (5.1)	2 (4.8)	0.933	
Chemotherapy drugs (%)	2 (2.1)	0 (0)	0.351	
Corticosteroids use within 28d (%)	3 (3.1)	2 (4.8)	0.619	
Mean CURB-65 score (SD)	2.5 (0.6)	2.4 (0.6)	0.612	
Neutropenia at influenza diagnosis (%)	1 (1.0)	1 (2.4)	0.534	
Lymphopenia at influenza diagnosis (%)	39 (39.8)	28 (66.7)	0.004*	3.03
Influenza type				
Influenza A (%)	86 (87.8)	39 (92.9)	0.371	
Influenza B (%)	12 (12.2)	3 (7.1)	0.371	

Data are displayed as mean (SD), or n (%). *p<0.05 vs Non-IAPA group. IAPA, influenza-associated pulmonary aspergillosis; Non-IAPA, influenza infection without pulmonary aspergillosis; COPD, chronic obstructive pulmonary disease. Neutropenia is neutrophil count ≤ 0.5×10^9^/L. lymphopenia is lymphocyte count ≤1×10^9^/L. OR, odds ratio.

### Diagnosis and management of IAPA

Non-specific respiratory symptoms such as cough, sputum production, fever, hemoptysis, chest pain, or dyspnea were present to varying degrees in both IAPA and Non-IAPA patients and did not have differential significance (Toda et al., 2021). It is worth noting that most IAPA patients in the present study do not show improvement in their respiratory symptoms before receiving antifungal treatment, or their symptoms worsen after a period of improvement. In this study, five to seven days after admission, patients who still had worsening respiratory symptoms with or without fever received chest CT examination timely. If IAPA or other con-infection was suspected, serum 1,3-β-D-glucan (G) test, GM test and bronchoscopy examination were performed. The obtained BALF samples were immediately subjected to microbiology rapid on-site evaluation (M-ROSE), including Gram staining, fungal smear, and acid fast staining. Culture, GM test and targeted next generation sequencing (tNGS) in BALF were also performed. If fungal staining of BALF reveals mold or GM test was positive and the probable IPA diagnosis was reached after evaluation, immediate antifungal treatment was initiated. If *Aspergillus* is detected in BALF samples through tNGS test or identified as *Aspergillus* after culture, and the probable IPA diagnosis was reached after evaluation, antifungal treatment will also be initiated. The criteria for elevated GM in serum or BALF: single sample ≥ 1.0, or serum ≥0.7 and BALF ≥0.8 (Donnelly et al., 2020).

### The chest CT characteristics of IAPA

**Figure 1:**
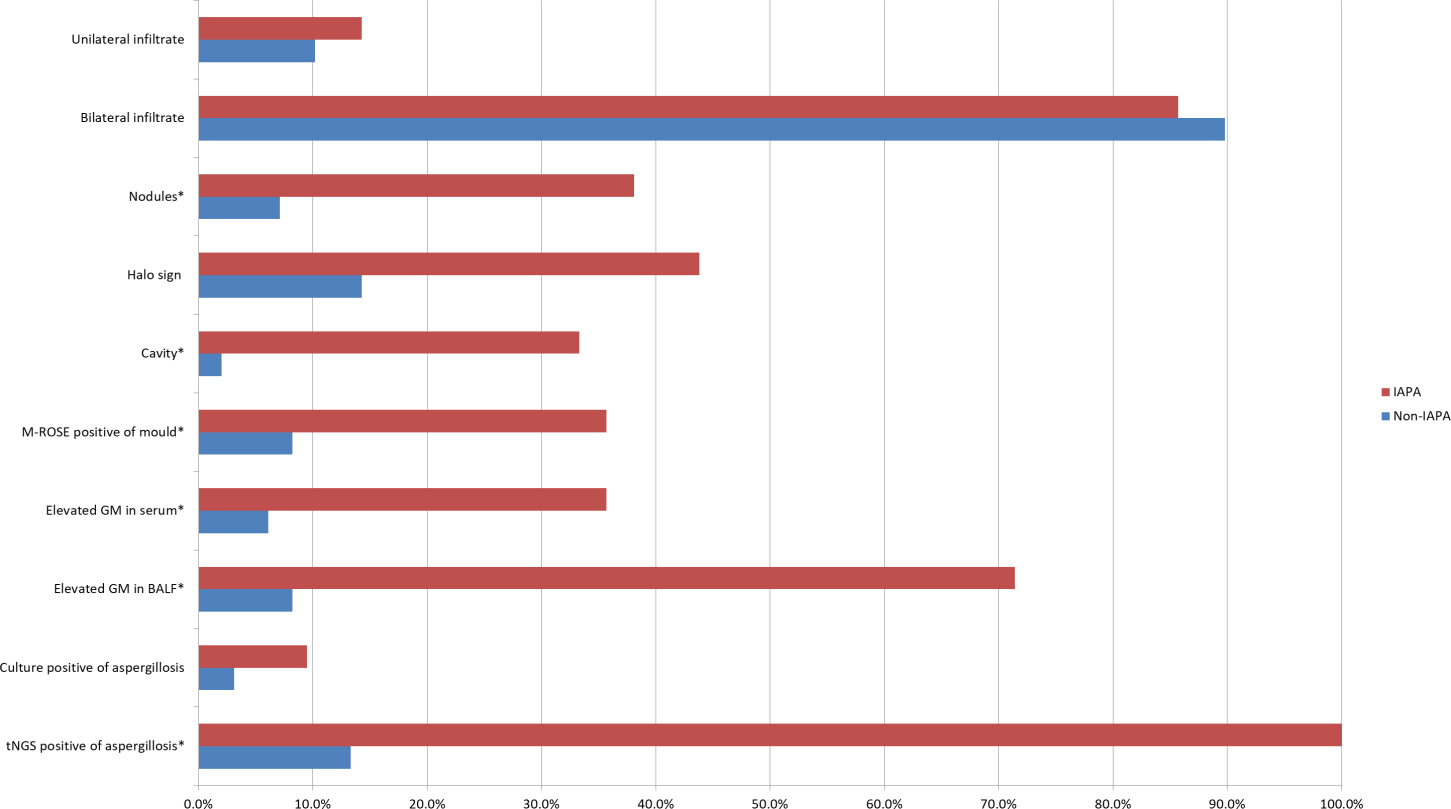
This bar chart is a visual representation of the diagnostic evidence data in **Table 2**. Data are displayed as %. *p<0.05 vs Non-IAPA group. IAPA, influenza-associated pulmonary aspergillosis; Non-IAPA, influenza infection without aspergillosis; M-ROSE, microbiology rapid on-site evaluation; GM, galactomannan; BALF, bronchoalveolar lavage fluid; tNGS, targeted next generation sequencing.

**Figure 2:**
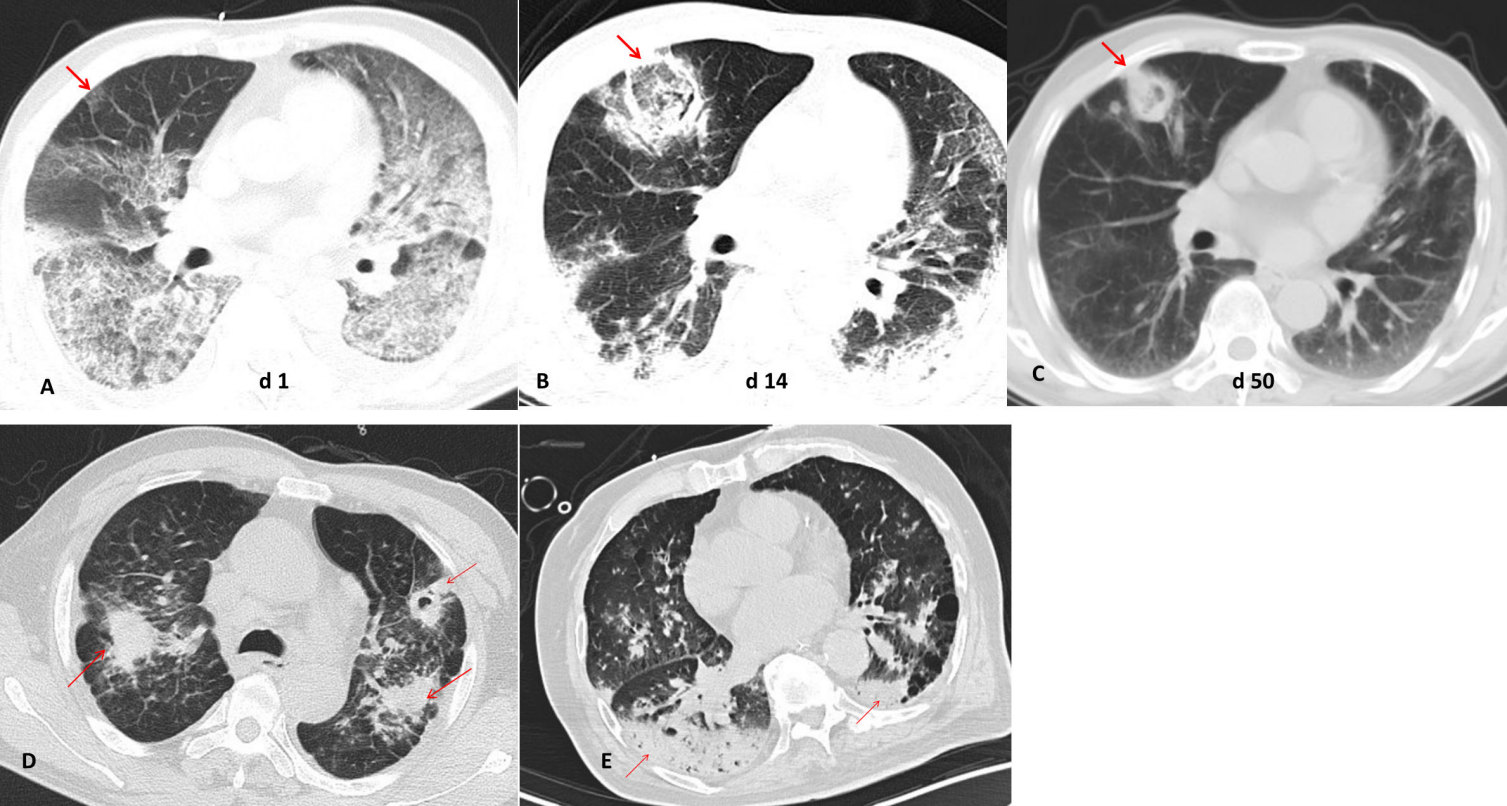
Different chest CT imagings in IAPA patients. **(A)** An IAPA patient’s right lung lesion initially presented as ground glass opacities, **(B)** then developed into subpleural wedge-shaped opacities, **(C)** and later became solid nodular opacities. **(D)** Multiple chest CT imaging forms such as cavities and consolidation shadows coexist in one IAPA patient. **(E)** Consolidation shadows with or without inflation sign in chest CT imaging of one patient. The red arrow indicates the lesion.

IPA often appears as wedge-shaped lung consolidation, multiple small nodules, masses, and multiple small patches on chest CT scans, and the most distinctive features are wedge-shaped consolidation shadows and small nodular edges with “halo sign”. These radiographic features are frequently nonspecific and can change as the disease progresses (Alexander et al., 2021). In this study, there was no difference in the distribution of lung lobes (unilateral, bilateral) between IAPA patients and the control group. The number of nodules and cavities in the IAPA group was significantly higher than that in the Non-IAPA group, although there were also more halo signs in IAPA patients, the difference was not statistically significant (**Table 2**; **Figure 1**). It is worth noting that the lung imaging of the same IAPA patient may have different manifestations at different stages of the disease course. As shown in **Figures 2A–C**, an IAPA patient’s right lung lesion initially presented as ground glass opacities, then developed into subpleural wedge-shaped opacities, and later became solid nodular opacities. In addition, there may not be only one chest CT manifestation in the same IAPA patient, and multiple imaging forms often coexist, such as cavities and consolidation shadows (**Figure 2D**), consolidation shadows with or without inflation sign (**Figure 2E**). When influenza patients present with unexplained imaging manifestations of viral infection in the lungs, timely and comprehensive examinations should be conducted to determine whether there is IPA, in order to avoid missed diagnosis.

**Table 2:** Chest CT and microbiology evidences for IAPA diagnosis.

Clinical features	Non-IAPA (*n* =98)	IAPA (*n* =42)	*p va*lue
Findings on chest CT			
Unilateral infiltrate (%)	10 (10.2)	6 (14.3)	0.487
Bilateral infiltrate (%)	88 (89.8)	36 (85.7)	0.487
Nodules (%)	7 (7.1)	16 (38.1)	0.000*
Halo sign (%)	1 (14.3)	7 (43.8)	0.172
Cavity (%)	2 (2.0)	14 (33.3)	0.000*
M-ROSE positive of mould (%)	8 (8.2)	15 (35.7)	0.000*
GM test			
Elevated GM in serum (%)	6 (6.1)	15 (35.7)	0.000*
Elevated GM in BALF (%)	8 (8.2)	30 (71.4)	0.000*
Culture positive of aspergillosis (%)	3 (3.1)	4 (9.5)	0.108
tNGS positive of aspergillosis (%)	13 (13.3)	42 (100)	0.000*

Data are displayed as n (%). *p<0.05 vs Non-IAPA group. IAPA, influenza-associated pulmonary aspergillosis; Non-IAPA, influenza infection without aspergillosis; M-ROSE, microbiology rapid on-site evaluation; GM, galactomannan; BALF, bronchoalveolar lavage fluid; tNGS, targeted next generation sequencing.

### The role of M-ROSE in IAPA diagnosis

**Figure 3:**
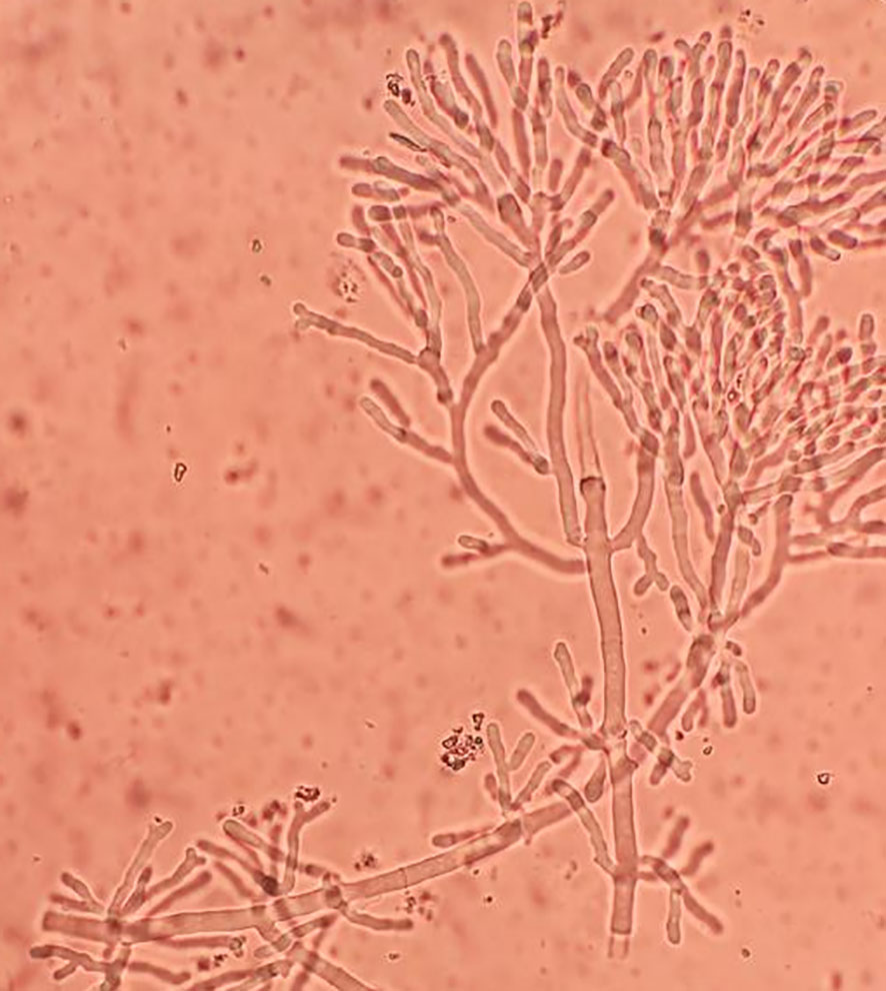
The presence of septate hyphae resembling antlers suggests an aspergillosis infection.

Early and rapid diagnosis of IPA has always been a goal pursued by clinical workers, but so far, there has been no breakthrough due to operability and technical difficulties. In this study, we used a microbiological rapid on-site evaluation (M-ROSE) method to perform wet slide examination of lower respiratory tract specimens, which can quickly provide some special and important information, especially for the rapid diagnosis of lower respiratory tract fungal infections (invasive aspergillosis, mucormycosis, etc.). The presence of septate hyphae resembling antlers suggests an aspergillosis infection (**Figure 3**), while the absence of branches or few branching aseptate hyphae may indicate a mucormycosis infection. **Table 2** shows that the probability of detecting hyphae using M-ROSE technology in IAPA patients reached 35.7% (15/42), significantly higher than that in the Non-IAPA group. Moreover, these patients who were detected hyphae in BALF under the microscope and were preliminarily judged to be *Aspergillus* based on morphology were ultimately confirmed by culture or tNGS results, demonstrating extremely high specificity. Therefore, based on clinical symptoms and chest imaging, patients suspected of having IPA should initiate antifungal treatment if their BALF was highly suspected of having *Aspergillus* through M-ROSE by experienced laboratory experts. And then further evaluation should be conducted based on tNGS, culture or histopathology results.

### The role of GM in IAPA diagnosis

The GM test plays a critical role in the diagnosis of IPA (Patterson et al., 2016; Sarrafzadeh et al., 2010). In immunocompromised patients, serum GM test demonstrates moderate sensitivity (69.2%) and specificity (72.2%) for IPA (Sarrafzadeh et al., 2010). A previous study showed that BALF GM test outperforms serum GM, with sensitivity reaching 91.7% and specificity 92.5 (Wu et al., 2021). In the present study, the sensitivity and specificity of serum GM for IAPA diagnosis were 35.7% (15/42) and 93.9% (92/98), respectively. As for BALF GM, the sensitivity and specificity were 71.4% (30/42) and 91.8% (90/98), respectively (**Table 2**; **Figure 1**). These results showed that BALF GM test significantly increased diagnostic sensitivity of IAPA.

### The culture in IAPA diagnosis

Microbial culture is crucial for diagnosing pathogens, however, the positive rate of *Aspergillus* culture is relatively low. Niu et al. reported that in patients with acute exacerbation of chronic obstructive pulmonary disease complicated with IPA, the positive rate of *Aspergillus* culture was only 29.0% (Niu et al., 2024), while it was even lower to 9.5% (4/42) in IAPA patients in the present study (**Table 2**; **Figure 1**).

### The tNGS in IAPA diagnosis

In the EORTC/MSGERC (2020) guideline, PCR technology was first recommended for the diagnosis of IPA (Donnelly et al., 2020). With the advancement of gene sequencing technology, many detection techniques based on PCR technology such as tNGS have made significant progress. The tNGS can detect dozens to hundreds of known pathogenic microorganisms in test samples through the combination of ultra-multiplex PCR amplification and high-throughput sequencing techniques (Wang et al., 2025). It showed in the present study that the sensitivity and specificity of tNGS for IAPA diagnosis were 100% (42/42) and 86.7% (85/98), respectively (**Table 2**; **Figure 1**).

### Co-infections pathogens of patients

**Figure 4:**
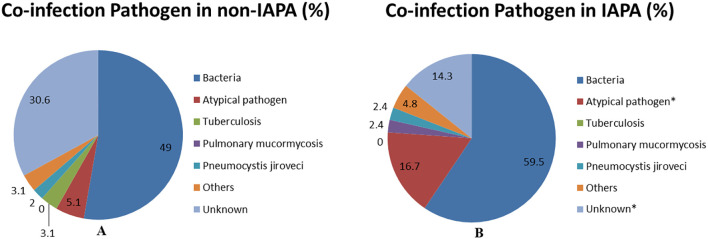
This pie chart is a visual representation of the co-infections data of non-IAPA **(A)** and IAPA **(B)** in **Table 3**. Data are displayed as %. *p<0.05 vs Non-IAPA group. IAPA, influenza-associated pulmonary aspergillosis; Non-IAPA, influenza infection without pulmonary aspergillosis.

Previous studies showed that co-infections were common in patients with influenza (Al-Dorzi et al., 2024; Fu et al., 2025). Similar to previous studies, bacterial co-infection accounts for the vast majority of co-infected pathogens in patients with influenza in this study. Bacterial co-infection​​ rates were numerically higher in IAPA patients (59.5%, 25/42) but did not reach statistical significance compared to Non-IAPA patients (49.0%, 48/98; p = 0.252). Different from those studies, we reported that atypical pathogens​​ (e.g., Mycoplasma, Chlamydia, Legionella) were frequently identified in the present study and they were more in IAPA patients (16.7%, 7/42) than those in Non-IAPA patients (5.1%, 5/98; p = 0.025). ​​*Mycobacterium tuberculosis*, were relatively rare and there was no significant difference between the two groups. Rare co-infections, such as ​​pulmonary mucormycosis​​ and Pneumocystis jirovecii, showed no significant differences. ​​Unknown pathogens​​ were less common in IAPA patients (14.3%, 6/42) than in Non-IAPA patients (30.6%, 30/98; p = 0.043) (**Table 3**; **Figure 4**).

**Table 3:** Co-infections pathogens in Non-IAPA and IAPA patients.

Clinical features	Non-IAPA (*n* =98)	IAPA (*n* =42)	*p va*lue
Bacteria (%)	48 (49.0)	25 (59.5)	0.252
Atypical pathogen (%)	5 (5.1)	7 (16.7)	0.025*
Tuberculosis (%)	3 (3.1%)	0 (0)	0.252
Pulmonary mucormycosis (%)	0 (0)	1 (2.4)	0.125
Pneumocystis jiroveci (%)	2 (2.0)	1 (2.4)	0.899
Others (%)	3 (3.1)	2 (4.8)	0.619
Unknown^#^	30 (30.6)	6 (14.3)	0.043*

Data are displayed as n (%). *p<0.05 vs Non-IAPA group. IAPA, influenza-associated pulmonary aspergillosis; Non-IAPA, influenza infection without pulmonary aspergillosis. ^#^ suspected respiratory infection and antibiotics were used, but no relevant pathogen was detected.

### Antifungal treatment of IAPA and outcome

For the treatment of IAPA, voriconazole was chosen as first-line drug (78.6%). For patients with severe liver or kidney dysfunction or intolerance, isavuconazole was given for treatment. In the IDSA (2016) guideline, the treatment duration for IPA should be continued for a minimum of 6–12 weeks, depending on the degree and duration of immunosuppression, site of disease, and evidence of disease improvement (Patterson et al., 2016). However, the average antifungal treatment duration (32.2 ± 4.8 d) in the present study was obviously shorter than that mentioned in the IDSA guideline (**Table 4**). These results suggest that IAPA patients improved faster than those with hematological disorders (Lanternier et al., 2020), and therefore the treatment duration for IAPA patients was shorter. As shown in **Table 4**, stay in hospital and 60-d mortality in IAPA group were greatly higher than those in Non-IAPA group, which suggested that co-infected with aspergillosis significantly increased stay in hospital and mortality.

**Table 4:** Antifungal treatment of IAPA and outcome.

Clinical features	Non-IAPA (*n* =98)	IAPA (*n* =42)	*p va*lue
Antifungal drugs			
Voriconazole (%)	0 (0)	33 (78.6)	
Isavuconazole (%)	0 (0)	9 (21.4)	
Mean antifungal drugs duration, d (SD)	0 (0)	32.2 (4.8)^#^	
Mean stay in hospital, d (SD)	6.8 (1.6)	13.2 (1.4)	0.000*
60-d mortality (%)	3 (3.1)	10 (23.8)	0.000*
Mean NLR (SD)	8.1 (5.8)	12.0 (10.1)	0.024*

IAPA, influenza-associated pulmonary aspergillosis; Non-IAPA, influenza infection without aspergillosis; NLR, neutrophil to lymphocyte ratio. ^#^Data of 32 survivals. *p<0.05.

### Prediction of IAPA prognosis

**Figure 5:**
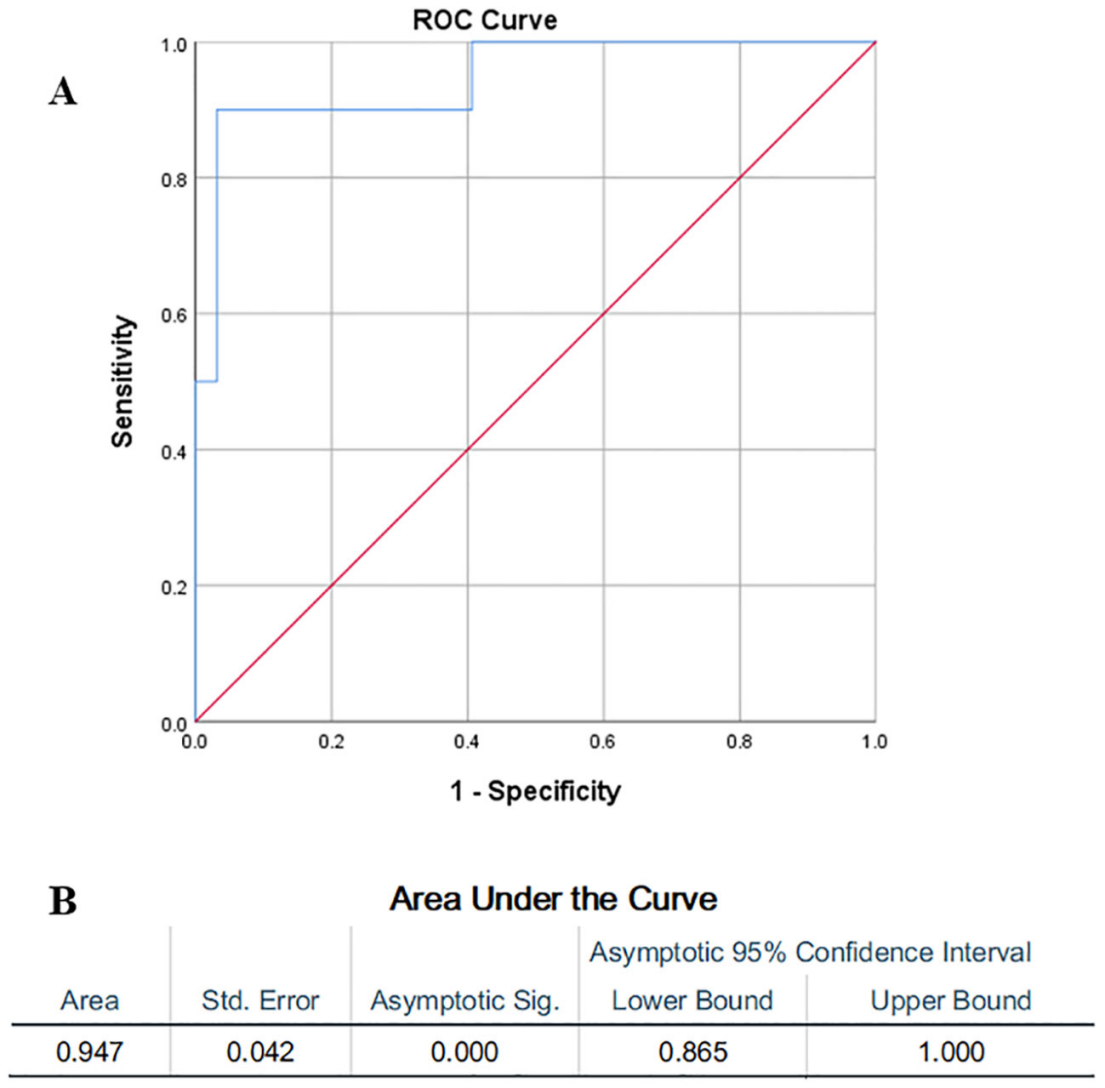
**(A)** The ROC curve analysis showed the value of NLR in predicting IAPA prognosis and **(B)** the results showed that the AUC was 0.95 (95% CI, 0.87–1.0). The optimal cutoff value of NLR was 17.70, corresponding to a sensitivity of 0.87 and a specificity of 0.97.

Neutrophil-to-lymphocyte ratio (NLR) is calculated using the absolute counts of neutrophils and lymphocytes obtained from routine complete blood counts. It is an inflammatory index reflecting systemic inflammatory cascades, which was considered to be a novel biomarker in patients with inflammatory diseases (Wu et al., 2024). A previous study showed that NLR is an important indicator of systemic inflammation (Zahorec, 2001). Whether NLR could predict IAPA prognosis remains unclear. As shown in **Table 4**, the average NLR in IAPA group was significantly higher than that in Non-IAPA patients. The ROC curve analysis result showed that the AUC was 0.95 (95% CI, 0.87–1.0), which indicated a high diagnostic accuracy (**Figure 5**). According to the optimal Youden index in ROC curve (Yang et al., 2025), the optimal cutoff value of NLR was 17.70, corresponding to a sensitivity of 0.87 and a specificity of 0.97. These results indicated that NLR has significant value in predicting the prognosis of IAPA.

## Discussion

Since the development of diagnostic and treatment guidelines for invasive fungal diseases by EORTC/MSGERC in 2020 (Donnelly et al., 2020), it played an important role in guiding the diagnosis and treatment of pulmonary fungal diseases. Although the EORTC/MSC guidelines list host factors that are prone to IPA, clinical practitioners have found in clinical practice that some risk factors, such as influenza, can also lead to IPA (Schauwvlieghe et al., 2018), even though influenza is not included in the host factors listed in the EORTC/MSGERC guideline. In order to further understand the characteristics of IAPA and based on our clinical practice, we retrospectively analyzed the risk factors, diagnostic process, treatment, and prognostic features of IAPA cases. In this study, we found that IAPA had the following characteristics: firstly, the vast majority of IAPA occurred within one week after hospitalization. Second, among the risk factors for influenza patients to develop IPA, Diabetes and lymphopenia were important risk factors. Thirdly, pulmonary imaging showed that IAPA patients had more nodular lesions in the lungs, and a higher proportion of them were accompanied by cavitary lesions. Fourthly, in terms of microbiological testing, the GM test in BALF showed high sensitivity and specificity for diagnosing IPA. The positive rate of *Aspergillus* culture was relatively low, while rapid pathogen diagnosis technique, M-ROSE, could effectively detect mould with high specificity. The tNGS, based on PCR technology and gene sequencing technology, was a new diagnostic technique for pathogenic microorganisms and had important value in detecting *Aspergillus*. Its sensitivity reached 100% and specificity reached 86.7% for detecting *Aspergillus* in the present study. These technologies each had their own advantages and complement each other. When used together, they could significantly improve the detection rate of *Aspergillus*. Fifthly, both IAPA and Non-IAPA patients had a higher rate of co-infections, with a significantly higher co-infection rate of atypical pathogens in the IAPA group compared to the Non-IAPA group. Sixth, in the treatment for IAPA, voriconazole was still used as the first-line medication, while esoconazole was used as an alternative for patients who could not tolerate voriconazole or had severe liver and kidney function impairment. The average treatment course for IAPA patients in the present study (32.2 ± 4.8d) was greatly shorter than the course specified in the IDSA (2016) guideline. This indicates that after antifungal treatment, IAPA patients’ condition improved quickly and did not require such a long course of treatment as specified in the IDSA (2016) guideline. Lastly, we found that NLR can predict the final prognosis of IAPA patients very well.

In previous guidelines for the diagnosis and treatment of IPA, host factors were mainly focused on immunocompromised populations, such as AIDS, hematological diseases, solid organ transplantation, etc (Donnelly et al., 2020; Patterson et al., 2016). In recent years, with the continuous deepening of research, the researchers found that there were some other risk factors that can lead to IPA in non-immune deficient populations, such as chronic obstructive pulmonary disease (COPD) and influenza (Schauwvlieghe et al., 2018; Waqas et al., 2020). Therefore, it may be necessary to include severe influenza as a risk factor for the occurrence of IPA.

It is worth noting that in this study, the proportion of Diabetes and Lymphopenia in IAPA group was significantly higher than that in Non-IAPA group, with OR values of 2.65 and 3.03 respectively, indicating that Diabetes and Lymphopenia were closely related to the occurrence of IAPA. The mechanism is that hyperglycemia can affect the function of innate and adaptive immune cells in the human body, weakening their anti-*Aspergillus* activity (Holt et al., 2024). The decrease in lymphocytes caused by influenza virus infection directly reduces the anti-*Aspergillus* effect of adaptive immunity (Jolink et al., 2017).

Early diagnosis and appropriate treatment of IAPA are crucial for improving the prognosis, and microbiological evidence is a key element in diagnosing IAPA. The sensitivity and specificity of traditional GM test are sometimes affected by many factors, which may result in false positives or false negative (Paiva and Pereira, 2019), while culture requires at least 3–5 days and has a lower positivity rate. M-ROSE technology has been used to guide antimicrobial therapy for critically ill patients (Tao et al., 2022). In this study, our team attaches great importance to the application of M-ROSE technology, although its detection sensitivity was not high, its specificity was very high. Once characteristic fungal hyphae are discovered, it has important diagnostic value. Moreover, this technology is simple and fast to operate. So it is worthy of clinical promotion. The tNGS technology for detecting *Aspergillus* has high sensitivity and specificity. Previously, its clinical application was limited by its high price. With the advancement of technology, the price has greatly decreased, and it has significant value for critically ill and undiagnosed patients. It should be pointed out that when it was positive for *Aspergillus* in BALF culture or tNGS test, it is needed to comprehensively judge whether it is *Aspergillus* infection based on the patients’ clinical manifestations, GM test results, chest CT imaging, and response to other treatments. As shown in **Table 2**, 3.1% of non-IAPA patients had culture positive and 13.3% had tNGS positive for *Aspergillus*. Although the BALF culture or tNGS were positive for *Aspergillus* in these patients, their GM test results and chest CT imaging were not consistent with IPA and they ultimately recovered after antibacterial treatments. So it was *Aspergillus* colonization in these patients. A survey conducted by Perfect in 2001 showed that only 12.2% of patients with positive *Aspergillus* culture (81% of whom were airway specimens) were ultimately diagnosed with IPA (Perfect et al., 2001), which showed a high proportion of *Aspergillus* colonization.

Co-infections were common in inpatients in the present study, and most of them were bacteria with no significant difference between both groups. Atypical pathogens in IAPA group were detected more than those in Non-IAPA patients, which indicated that the decrease in lymphocytes led to a significant reduction in their ability to kill intracellular parasitic bacteria such as atypical pathogens (Jones and Simecka, 2003).

In previous guidelines, voriconazole was recommended as the first-line drug for treating IPA, and later on, esoconazole and posaconazole were added (Epelbaum et al., 2024). The current guidelines do not recommend routine use of combination therapy for IPA, however, the combination therapy may have certain benefits for patients undergoing hematopoietic stem cell transplantation, critically ill patients and in the area with high triazole resistance (Epelbaum et al., 2024). Although this is a multicenter study, it has certain limitations due to its retrospective nature. It is necessary to conduct large-scale multicenter prospective studies in the future to further explore the epidemiological and clinical characteristics, clinical features, different microbial diagnostic techniques, and treatment options for IAPA.

## Conclusions

In conclusion, it showed in the present study that Diabetes and lymphopenia were important risk factors for IAPA. The comprehensive application of serum and BALF GM, M-ROSE and tNGS could significantly improve the detection rate of *Aspergillus*. The treatment duration for IAPA was ~32.2 d, which was obviously shorter than the course mentioned in the IDSA (2016) guideline. The ROC curve analysis showed that NLR could effectively predict the prognosis of IAPA.

## Data Availability

The original contributions presented in the study are included in the article/supplementary material. Further inquiries can be directed to the corresponding authors.
